# Leptin gene polymorphism affects leptin level in childhood asthma

**DOI:** 10.1007/s12519-018-0182-2

**Published:** 2018-09-10

**Authors:** Dawid Szczepankiewicz, Paulina Sobkowiak, Beata Narożna, Irena Wojsyk-Banaszak, Anna Bręborowicz, Aleksandra Szczepankiewicz

**Affiliations:** 1 Department of Animal Physiology and Biochemistry, Poznan University of Life Sciences, Poznan, Poland; 2 Department of Pediatric Pulmonology, Allergy and Clinical Immunology, IIIrd Department of Pediatrics, Poznan University of Medical Sciences, Poznan, Poland; 3 Laboratory of Molecular and Cell Biology, Department of Pediatric Pulmonology, Allergy and Clinical Immunology, IIIrd Department of Pediatrics, Poznan University of Medical Sciences, Szpitalna St. 27/33, 60-572 Poznan, Poland

**Keywords:** Asthma, Gene, Leptin, Leptin receptor, Polymorphism

## Abstract

**Background:**

Leptin may induce inflammation in asthma by activation of Th2 cells. It has also been demonstrated that leptin expression increases upon inflammation and that asthmatic patients show increased serum leptin levels. We hypothesized that the polymorphism in leptin (*LEP*) and leptin receptor (*LEPR*) genes is associated with childhood asthma and may affect their serum level. To our knowledge, there are no reports analyzing *LEP* and *LEPR* polymorphisms in association with their serum levels in childhood asthma.

**Methods:**

We analyzed 35 subjects: 25 asthmatic pediatric patients and 10 healthy children aged from 6 to 18. The diagnosis of allergic asthma was based on clinical manifestation, lung function, positive skin prick tests and increased immunoglobulin E levels. The polymorphisms were genotyped with use of polymerase chain reaction-restriction fragment length polymorphism method. Serum levels of leptin and leptin receptor were determined using BioVendor enzyme-linked immunosorbent assay kits. Statistical analysis was done with Statistica v.12.

**Results:**

We observed that leptin levels were increased in asthmatic subjects as compared to healthy controls and were significantly higher during exacerbation than in the asymptomatic period (*P* = 0.025). We observed that *LEP* polymorphism (rs13228377) was associated with higher serum leptin levels in asthma and these two variables had high predictive value for asthma risk (*P *= 0.007, odds ratio 17.5, predictive accuracy 83.9%). *LEPR* polymorphisms did not show association with its serum level and asthma risk.

**Conclusion:**

*LEP* polymorphism may increase asthma risk via influence on its serum level.

## Introduction

Asthma is the most common chronic disease of childhood. It is characterized by both local inflammation in the airways as well as low grade systemic inflammation. One of the peptides involved in various inflammatory diseases is leptin, a hormone that belongs to the interleukin 6 cytokine family. It is mainly produced by adipose tissue and exerts its neuroendocrine effects in the control of food intake and energy expenditure in hypothalamus. In addition to the hypothalamus, leptin binding has also been reported in hematopoietic cells, liver, kidney, skin, stomach, heart, spleen and lung tissue. In the lung, leptin, upon binding to its receptor, shows a pro-inflammatory effect [1–3]. In response to inflammatory stimuli, increased leptin mRNA and protein levels have been observed and are associated with symptoms intensification and exacerbation [4, 5].

In animal studies, leptin deficiency decreased inflammatory response in autoimmune diseases [6, 7]. Moreover, models using genetically modified mice sensitized with ovalbumin showed that leptin led to pathological increase in airways reactivity and enhanced cytokine production in response to ozone in comparison to control mice [8].

Recent in vitro studies also showed that viral infection, the main cause of asthma exacerbations, led to increased secretion of leptin by bronchial epithelial cells resulting in activation of Th17 cells differentiation, and inhibition of Th2 cells differentiation [9]. These findings were confirmed by another in vitro study showing that leptin enhanced airway inflammation via increased intercellular adhesion molecule-1 expression and migration as well as cytokine synthesis in the human airway epithelium [10].

Previous clinical studies showed increased leptin levels in serum of adult patients diagnosed with asthma [11] and in children with atopic asthma as compared to healthy control groups [12, 13]. The reported increased leptin was independent of body mass index (BMI) value, thus suggesting that it may play a role in asthma independent of obesity.

It seems that increased leptin level is specific for asthma, in particular, those with severe symptoms. This hypothesis was confirmed by a recent study showing increased leptin levels in severe asthma [14]. These results suggested that leptin may be used as a biomarker of severe asthma endotype characterized by lower lung function, systemic inflammation and more severe respiratory symptoms [14].

Leptin levels were also associated with the severity of clinical symptoms as reported by Unal et al. [15] who showed increased leptin level in patients with allergic rhinitis patients during symptomatic period, although no association was observed with allergic rhinitis without asthma [13].

Leptin exerts its biological role via Ob-R transmembrane receptor in the hypothalamus. This receptor is also present on the surface of structural cells (epithelium, smooth muscle, fibroblasts) in the lungs. The different isoforms participate in leptin signal transduction, although the exact role of soluble form is not well understood. So far, no study analyzing the role of leptin receptor gene (*LEPR*) polymorphism on leptin receptor serum level in asthma has been performed.

In our previous study, we reported that childhood asthma is associated with polymorphism in leptin gene (*LEP*), but not with *LEPR* [16]. Based on the above, we hypothesized that serum leptin level may be influenced by genetic polymorphism in *LEP* as well as by clinical condition in asthmatic patients. We also aimed to analyze if leptin receptor level or gene polymorphism may influence leptin level and asthma risk.

## Methods

### Patients

The study included 25 asthmatic patients of Caucasian origin: 6-18 years old [mean age 9.77 years, standard deviation (SD) 3.73]. Patients were recruited from hospitalized patients, treated for asthma in the Department of Pulmonology, Pediatric Allergy and Clinical Immunology, Poznan University of Medical Sciences. The asthma diagnosis was made according to GINA 2002 recommendation, based on clinical asthma symptoms and lung function testing. Clinical diagnosis of atopy depended on current or past symptoms of atopic dermatitis and/or allergic rhinitis. Atopy was confirmed with total immunoglobulin E (IgE) level higher than the upper normal limits for age and positive skin prick test to at least one aero-allergen (*Dermatophagoides pteronyssinus*, *Dermatophagoides farinae*, cat, dog, feathers, *Alternaria alternata*, *Cladosporium herbarum*; pollen: grass mix, rye, birch pollen, alder, hazel–Allergopharma, Germany).

The patients were analyzed twice: during exacerbation of symptoms and in the asymptomatic period. Exacerbation was defined as the presence of asthma symptoms (daytime symptoms, nocturnal symptoms, limitation of daily activities, the need for medical treatment, and reduced lung function) due to allergen exposure or respiratory viral infection. Asymptomatic period was characterized by good control of asthma symptoms and no acute infections for 2 weeks before blood collection.

### Control group

Control group consisted of 10 healthy subjects of Caucasian origin (mean age 12.6 years, SD = 3.02). Control subjects were also recruited from the same geographic region from the group of carefully chosen volunteers without asthma and allergy symptoms. Any allergic diseases or asthma were excluded based on clinical examination, spirometry and exhaled NO measurement in the airways. We also excluded individuals with chronic diseases other than allergy and asthma.

All participants as well as their parents had given written informed consent. The project was approved by our local ethics committee accepted. Study was performed in compliance with the Code of Ethics of the World Medical Association (Declaration of Helsinki).

### Genotyping

The DNA was extracted from 10 mL of ethylene diamine tetraacetic acid anticoagulated whole blood using the salting out method [17]. We analyzed two *LEP* polymorphisms: upstream of coding gene region: rs13228377 (A/G) and rs2167270 (-2549T/G), and two *LEPR* polymorphisms: rs1137100 (Lys109Arg), rs12131454 (Glu223Arg). The polymerase chain reaction-restriction fragment length polymorphism analysis was done as described previously [16].

### Enzyme-linked immuno sorbent assay (ELISA)

Blood samples were collected using sample tubes without anticoagulant. After 1 hour, the sera were collected by centrifugation, and stored frozen at − 80 °C until further analysis. Concentration of leptin and leptin receptor in undiluted serum samples was measured using ELISA kit (BioVendor, Czech Republic) according to the manufacturer’s protocol.

### Statistical analysis

The Pearson’s Chi square (*χ*^2^) and Fisher’s exact tests were used to analyze differences in genotypic and allelic distribution between patients and control subjects. Normality of data distribution was tested using Shapiro–Wilk test. Differences between groups were analyzed using ANOVA analysis of variance. Within-group differences were evaluated with paired *t* test. Stepwise logistic regression analysis with Wald test was performed using disease status (case–control) as a dichotomous-dependent variable and the following predictors included in the model: genotypes of *LEP* and *LEPR* polymorphisms and serum leptin and leptin receptor levels. Statistical analyses were performed using Statistica v.12.0 software (Statsoft Polska). Statistically significant level was below 0.05.

## Results

Asthmatic patients and control subjects included in the study did not differ significantly in gender and BMI, but asthmatic patients were younger than the healthy subjects (Table 1). Lung function results did not differ significantly between control group and asthmatic patients during asymptomatic period, but showed a significant decline during asthma exacerbation. The patients also demonstrated significant differences in total IgE levels as compared to the controls (Table 1).

**Table 1:** Clinical description of the analyzed population

Variables	Patients group (*n* = 25)	Control group (*n* = 10)	*P*
Male, %	53.80	50.00	0.87
Age (y), mean ± SD	9.95 ± 3.47	12.60 ± 3.02	0.04
BMI, mean ± SD	18.16 ± 3.08	20.17 ± 2.30	0.62
FEV_1_/FVC pred, mean ± SD	97.95 ± 8.96	98.40 ± 4.65	0.88
FEV_1_% pred, mean ± SD	100.10 ± 11.75	103.30 ± 13.61	0.49
FVC% pred, mean ± SD	93.50 ± 22.02	98.60 ± 14.80	0.50
FEV_1_/FVC pred exacerbation, mean ± SD	87.50 ± 14.90	–	
FEV_1_% pred exacerbation, mean ± SD	82.25 ± 19.58	–	
FVC% pred exacerbation, mean ± SD	88.00 ± 14.85	–	
IgE (IU/mL), mean ± SD	257.5 ± 326.7	7.32 ± 12.66	0.02

*BMI* body mass index, *FEV*_*1*_ forced expiratory volume in 1 s, *FVC* forced vital capacity, *IgE* immunoglobulin E, *SD* standard deviation

### Serum leptin and leptin receptor levels in atopic asthma

**Fig. 1:**
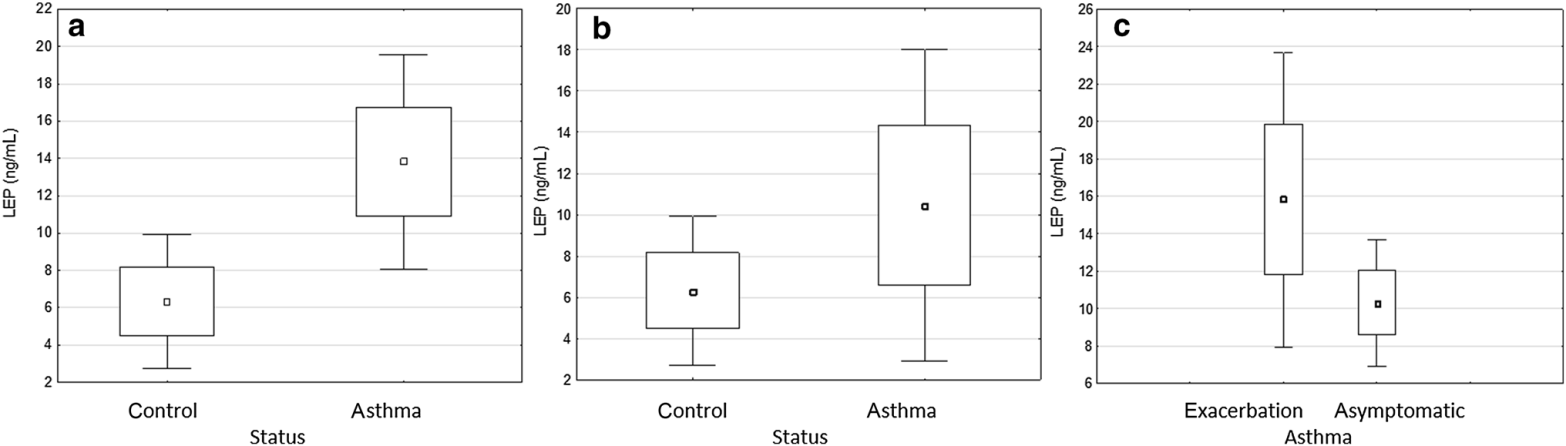
Comparison of leptin levels in serum between asthmatic patients during exacerbation and the control group (**a**); asthmatic patients during asymptomatic period and the control group (**b**); and asthmatic patients during exacerbation and in the asymptomatic period (**c**) (ANOVA, box plot: mean ± SE with whiskers: 1.96SE). *SE* standard error, *LEP* leptin

We observed that serum leptin level was higher in asthmatic patients during exacerbation (13.81 ± 10.56 ng/mL) than in the control subjects (6.32 ± 5.20 ng/mL), but this difference showed marginal significance (*P* = 0.078) (Fig. 1a). Asthmatic patients during asymptomatic period (control visit) also had higher leptin levels (10.46 ± 11.55 ng/mL) than the control group although the difference was not significant (*P* = 0.367) (Fig. 1b). However, analysis of leptin level between asthmatic patients with different clinical condition (exacerbation vs. stable period) showed significantly increased leptin level during asthma exacerbation (*P* = 0.025) (Fig. 1c).

Serum leptin receptor analysis showed that the mean LEPR level was 15.01 ± 5.68 ng/mL in patients during exacerbation versus 14.5 ± 5.38 ng/mL in control subjects and did not differ significantly (*P* = 0.191). Similarly, no significant difference was observed between patients in the stable period (16.40 ± 5.64 ng/mL) and healthy subjects (*P* = 0.719). Analysis of serum leptin receptor level between exacerbation and stable period also showed no significant difference (*P* = 0.207).

The analysis of serum leptin and leptin receptor levels did not correlate significantly with lung function measures, IgE levels and BMI values in asthmatic patients.

### Leptin and leptin receptor levels and genotypes

We found that 5′-UTR *LEP* polymorphism (rs13228377) correlates with serum leptin levels, with AA genotype related to increased leptin level independent of asthma status, although the difference was marginally significant (*P* = 0.08). We also found that the risk genotype (AA) was significantly more frequent in asthmatic patients than in the control group (*P* = 0.010). For the other analyzed *LEP* polymorphisms, we did not show significant differences in leptin serum levels.

Two analyzed *LEPR* polymorphisms (K109R and Q223R) did not affect significantly serum leptin receptor level independent of asthma status (*P* = 0.693 and *P* = 0.692, respectively). Moreover, no significant differences between asthmatic patients and controls were observed in genotype distribution for both *LEPR* polymorphisms (*P* = 0.072, *P* = 0.217, respectively).

### Leptin receptor and *LEPR* genotype

When we analyzed if two nonsynonymous polymorphisms in the *LEPR* gene may affect leptin binding in serum, we found that no difference was observed between the genotypes and leptin serum level (Table 2).

**Table 2:** Analysis of genotype of nonsynonymous *LEPR* polymorphism on leptin serum level

*LEPR* genotype	Mean LEP (ng/mL)	SD	*P*
K109R			
1	8.91	5.10	0.312
2	11.65	10.96	
3	2.13	0.62	
Q223R			
1	10.24	5.11	0.978
2	11.03	10.63	
3	10.28	11.78	

*LEPR* leptin receptor gene, *LEP* leptin, *SD* standard deviation

### Logistic regression analysis

To analyze if genotypes of *LEP* and *LEPR* polymorphisms together with their serum levels could be good predictors of asthma risk, we included them in the stepwise logistic regression analysis. We found that including all variables in the model is not specific for asthma (*P* = 0.230). Reducing number of variables to serum levels or genotypes separately also was not useful for asthma prediction (*P* = 0.570 and *P* = 0.126, respectively). However, taking into account an association of rs13228377 *LEP* polymorphism with asthma and higher leptin levels in asthma, we included them together in a predictive model. We observed that including these two variables significantly improved the model. The genotype of rs13228377 *LEP* polymorphism and serum leptin level significantly increased asthma risk (*P *= 0.008), showing odds ratio 16.7 and good predictive accuracy (overall 83.9%, in asthma 91.3%, in controls 62.5%).

## Discussion

The main finding of our study is an association of *LEP* polymorphism and serum leptin level with asthma. Our results confirmed previous association of the same *LEP* variant with atopic asthma in an independent cohort of pediatric patients.

Our previous study performed on *LEP* and *LEPR* gene polymorphism showed an association of rs13228377 polymorphism with childhood asthma with AA risk genotype and A risk allele in a cohort of Polish children [16]. The associated leptin polymorphism is localized upstream of the coding gene region, in the 5′-UTR of the *LEP*. This variant is within the regulatory gene region (CTCF binding site) and nucleotide substitution alters the regulation of gene transcription resulting in increased leptin levels in the carriers of AA risk genotype (according to Ensembl database). This is the first study that showed association of rs13228377 polymorphism with serum leptin level.

The interesting observation from our study is that leptin level is significantly increased during asthma exacerbation suggesting its role in active inflammatory processes. This is in line with the previous studies that showed increased secretion of leptin by epithelial cells infected with respiratory syncytial viral virus in vitro [9] and also in clinical setting that upon allergen challenge, leptin level was significantly higher during pollen season in seasonal allergic rhinitis patients than outside pollen season [18]. It was also shown that asthmatic children presented with increased serum leptin levels on admission, and the level decreased after 4 weeks of anti-inflammatory treatment [12]. Moreover, these authors observed that after treatment, serum leptin level was comparable to that seen in healthy control subjects.

In the recent genome wide association study (GWAS) performed in a cohort of over 32,000 participants [19], the authors analyzed thousands of chromosomal loci that may be responsible for circulating leptin levels. They found four loci, after adjusting for BMI, that were significantly associated with leptin levels: in/near *LEP*, *SLC32A1*, *GCKR*, *CCNL1*. More in-depth analysis revealed that common (MAF 49%) rs10487505 variant located 21 kb from *LEP* gene is in modest linkage disequilibrium (LD) (*r*^2^ = 0.4, *D*’ = 0.8) with the rs2167270 (A19G), the variant that has been extensively studied in candidate gene studies and leptin serum levels [20, 21]. Another study on serum leptin level reported an association of the same variant (rs10487505) with leptin level [22]. Analysis of LD showed that the previously analyzed variant (rs2167270) is in strong LD with the polymorphism analyzed in this study (rs13228377) (*D*’ = 1.0, *r*^2^ = 0.612). This may further support the observed association of rs13228377 with increased leptin level.

Leptin receptor plays an essential role in mediating the physiological effects of leptin. In the study by Sun et al. [23], the authors performed GWAS to identify single nucleotide polymorphisms (including two common nonsynonymous *LEPR* variants) associated with soluble form of the receptor; however, these *LEPR* variants were not associated with leptin receptor levels, which is in line with the results in our study. Previous reports also showed abnormally high circulating leptin receptor levels in carriers of rare *LEPR* mutations [24], but not polymorphisms. Moreover, the study by Quinton et al. demonstrated that Gln223Gln genotype was associated with lower leptin binding capacity to the soluble form of the receptor in plasma [25], indicating abnormal receptor function that may contribute to leptin resistance [26].

Taking into account previous reports, we investigated if two *LEPR* polymorphisms, K109R and Q223R, are associated with asthma risk or serum levels of leptin receptor or leptin. However, we did not find such associations This result is consistent with our previous study [16] and in line with the recent study by Kilpelainen et al. [19] who did not observe an association between *LEPR* variants and circulating leptin levels. Although two nonsynonymous *LEPR* variants are localized in the coding sequence of the gene and lead to aminoacid change in the extracellular domain of the receptor, no experimental data exist that could confirm their possible influence on receptor activity or function at the molecular level. To our knowledge, no other association studies of *LEPR* polymorphisms were performed in asthma so far, so we cannot compare our results. Leptin receptor levels were not previously described in association with childhood asthma, so it is difficult to verify our results.

The main limitation of this study is the small sample size. However, taking into account that this is a pilot study, we would like to emphasize that an association of rs13228377 polymorphism with asthma was previously observed also in an independent pediatric cohort (*n* = 243) [16]. Association of this variant with leptin serum level and lack of association between leptin receptor and its level in asthma were not published previously.

In conclusion, we report here that rs13228377 *LEP* polymorphism may affect transcription regulation of leptin gene and subsequently its serum level. However, leptin secretion stimulated upon asthma exacerbation suggests that also other than genetic regulatory elements influence its serum level during active airway inflammation process. Due to the limited sample size of this study, further analyses are warranted to verify our findings.
